# The Antioxidant Properties of Extracts of *Cuscuta* spp. Depend on the Parasite and the Host Species

**DOI:** 10.3390/antiox14070761

**Published:** 2025-06-20

**Authors:** Vanina Lozanova, Denitsa Teofanova, Bilyana Chakarova, Krasimir Rusanov, Kalina Pachedjieva, Anita Tosheva, Tzvetelina Zagorcheva, Lyuben Zagorchev

**Affiliations:** 1 Faculty of Biology, Sofia University “St. Kliment Ohridski”, 8 Dragan Tsankov blvd., 1164 Sofia, Bulgariateofanova@biofac.uni-sofia.bg (D.T.); atosheva@biofac.uni-sofia.bg (A.T.); 2 AgroBioInstitute, Agricultural Academy, 8 Dragan Tsankov blvd., 1164 Sofia, Bulgaria; bilyana.chakarova1@gmail.com (B.C.); krusanov@abv.bg (K.R.); tzvetelina.zagorcheva@gmail.com (T.Z.); 3 Centre of Competence “Sustainable Utilization of Bio-Resources and Waste of Medicinal and Aromatic Plants for Innovative Bioactive Products” (BIORESOURCES BG), 1000 Sofia, Bulgaria; 4 Research and Development and Innovation Consortium, Sofia Tech Park JSC, 111, Tsarigradsko Shose blvd., 1784 Sofia, Bulgaria

**Keywords:** antioxidants, bioactive compounds, flavonoids, parasitic plants, polyphenolics

## Abstract

Dodders (*Cuscuta* spp.) are prominent parasitic plants widely known and exploited in traditional medicine. They are rich in polyphenolics, which determine their strong antioxidant potential. However, comparatively few of the nearly 200 known species have been characterized for their medicinal potential. In the present study, we aimed to explore the antioxidant potential of four of the most widely distributed *Cuscuta* species in Bulgaria—*C. campestris*, *C. monogyna*, *C. epithymum*, and *C. europaea*. They differed significantly in polyphenolic content and accordingly differed in their antioxidant properties, although this correlation is not always straightforward, as shown in *C. europaeae*. Furthermore, we evaluated the host plant species’ influence on the polyphenolic content, antioxidant properties, and flavonoid profile of *C. campestris*, finding a significant enhancement when the parasite was grown on aromatic plants—rosemary and thyme—compared to a model host—*Arabidopsis thaliana*. Seven major flavonoids and phenolic acids—chlorogenic acid, kaempferol-3,7-*O*-diglucoside, quercetin-3-*O*-galactoside, kaempferol-3-*O*-galactoside, quercetin-3-*O*-glucoside, astragalin, and isorhamnetin-7-glucoside—were annotated after HPLC-MS analysis and found to be affected by the host species. In conclusion, it was found that extracts from different *Cuscuta* species differ in their antioxidant potential, which the host plants might further modify.

## 1. Introduction

Parasitic flowering plants are a widely variable group of nearly 4500 species, adapted to be partially (hemiparasites) or fully (holoparasites) dependent on their hosts for water, minerals, and organic nutrients [1]. Although traditionally regarded as harmful, very few represent serious agricultural pests, most notably several species of the *Striga*, *Orobanche/Phelipanche*, and *Cuscuta* genera [2]. Besides this negative impact, most of them are intriguing organisms with peculiar adaptations related to their semi- or fully non-photosynthetic lifestyle. They also play an important role in natural communities, regulating biodiversity by preferentially affecting particular plant groups, thus benefiting others [3,4]. Interestingly, most, if not all, are also employed in traditional medicine and are known to contain a large variety of bioactive compounds. Virtually all groups are known to possess medicinal properties, including members of the prominent families of broomrapes, Orobanchaceae [5]; mistletoes in the order Santalales, family Santalaceae [6]; and Loranthaceae [7], as well as some even more peculiar species like the “Maltese mushroom”, *Cynomorium coccineum* L. [8], and “the world’s largest flower”, *Rafflesia* spp. [9].

In addition to the abovementioned, members of the genus *Cuscuta* (family Convolvulaceae), or dodders, are also known for their use in traditional medicine. They are a diverse, widespread genus of about 200 species of stem (e.g., infecting the above-ground tissues of the host) and holoparasitic (e.g., depending fully on the host) plants [10]. Some, like *C. campestris* Yunck., are widespread agricultural pests [2], while others are rare or even threatened with extinction and need conservation efforts [11]. In Chinese traditional medicine, this genus is particularly appreciated for its medicinal properties and wide range of bioactivity. The most exploited species, *C. chinensis* Lam., locally known as Tu-Si-Zi, is sold as dried seeds and used as an extract as a tonic, improving sexual function and exhibiting positive effects on the immune system [12]. Ahmad [13] summarized a wide range of antibacterial, antifungal, antioxidant, anticancer, anti-inflammatory, hepatoprotective, and numerous other effects of about a dozen of *Cuscuta* spp., determined by hundreds of different bioactive compounds, including alkaloids, glycosides, steroids, terpenoids, carotenoids, organic acids, and (most notably) flavonoids. The antioxidant activity was found to be strongly correlated with the flavonoid content, especially quercetin and kaempferol [14]. While seeds are traditionally used, vegetative parts (e.g., stems) are equally rich in bioactive compounds [12].

An important question is whether all *Cuscuta* spp. are similar in the accumulation of bioactive compounds. Significant differences in flavonoids have been found between species, and they have even been proposed as chemotaxonomical markers [12,13]. In addition to the genetically determined species differences, the metabolic constituents in parasitic plants, and *Cuscuta* spp. in particular, could be affected by the host plant’s identity. Flores-Sanchez [15] concluded that at least some of the specialized metabolites in *Cuscuta* are transferred from the host through the haustoria (the parasitic organ, essentially a link between the vascular elements of the host and the parasite) and further modified in the parasite.

Recent reports suggest around nine or ten naturally occurring *Cuscuta* species in Bulgaria [16,17], of which at least some are listed as medicinal in the literature [18,19], with *C. europaea* L. being at the top of these lists. However, few studies have been conducted in this country [20] or globally [21]. Another prominent medicinal *Cuscuta* species that is not listed as such in Bulgaria but has been established in the country is *C. epithymum* L. [22]. As suggested by the recent literature, every *Cuscuta* species is a potential source of bioactive compounds. With the present study, we aimed to further explore the biology of these otherwise harmful and neglected parasitic plants in Bulgaria by focusing on their antioxidant properties and phenolic content as a pilot study on the potential for their sustainable utilization.

## 2. Materials and Methods

### 2.1. Plant Material

A total of 27 samples of *C. campestris*, 5 samples of *C. epithymum*, and 2 samples of *C. europaea*, which were previously described [23] and stored at −80 °C, were used in the present study. In addition, three samples of *C. monogyna* Vahl. collected in 2022 were also included in the analyses.

For laboratory-grown plant samples, seeds of *C. campestris* from an in-house sustained line (SU-BG-CA-2024.3) were treated according to a widely accepted method of sulfuric acid scarification. Germinated seedlings were used for the infection of fully grown *Arabidopsis thaliana* plants—Columbia ecotype, Col-0—and commercially available thyme (*Thymus vulgaris* L.) and rosemary (*Salvia rosmarinus* Spenn. (syn. *Rosmarinus officinalis* L.)) plants, all in triplicate. Parasite–host pairs were grown in glasshouse conditions for three weeks, which was sufficient to collect about 0.5–1 g of *Cuscuta* fresh mass from each host individual.

### 2.2. Extraction and Polyphenol Determination

The extraction of polyphenolic compounds was carried out by grinding 100 mg of vegetative mass in liquid nitrogen and adding 1 mL of 100% methanol (HPLC grade). After thorough homogenization, the material was centrifuged at 12,000× *g* for 10 min at 4 °C, and the resulting supernatant was separated for further analyses. Total polyphenols were measured by the Folin-Ciocalteu method [24], as employed by Thitilertdecha and Rakariyatham [25] with slight modifications. Briefly, 20 µL of the plant extract was mixed with 1.58 mL of dH_2_O, 0.1 mL of Folin reagent, and 0.3 mL of 1.8 M Na_2_CO_3_. Following incubation at 40 °C for 30 min in a heat block, absorbance at 765 nm was measured on a UV–Vis spectrophotometer (Jenway 6305), and polyphenol concentrations were calculated as gallic acid equivalents using a molar absorption coefficient of 1.075. Statistical significance was tested by Student’s *t*-test in GraphPad Prism version 8.0.0 (Boston, MA, USA).

### 2.3. ABTS Radical Scavenging Assay

The antioxidant activity of plant extracts was measured by the 2,2′-azino-bis(3-ethylbenzothiazoline-6-sulfonic acid (ABTS) radical scavenging assay according to Re [26]. Briefly, a solution of 7 mM ABTS and 2.45 mM K_2_S_2_O_8_ was left for 16 h at room temperature in the dark to allow for ABTS^•+^ formation. The resulting colored solution was diluted in dH_2_O until absorption of 0.7–0.8 (λ = 734 nm) was reached. In total, 0.9 mL of the ABTS^•+^ solution was mixed with 0.1 mL of plant extract in different dilutions, and absorption was measured at λ = 734 nm. Antioxidant activity was expressed as the percent inhibition of the ABTS radical using Equation (1), where A_b_ is the absorption of the blank (0.1 mL 100% methanol instead of plant extract), and A_s_ is the sample absorption.

(1) $$\%\;inhibition=\frac{Ab-As}{Ab}\cdot 100$$

### 2.4. HPLC-MS Analysis

HPLC-MS analysis was performed using a Shimadzu Nexera 40 HPLC system, equipped with a PDA and LC-MS-2050 mass detector (Shimadzu Corporation, Kyoto, Japan). Separation was performed using a Shimadzu Shim-pack Velox C18 (1.8 μm; 2.1 × 50 mm) chromatographic column at 28 °C temperature. The mobile phase consisted of 0.1% formic acid in water (A) and 0.1% formic acid in acetonitrile (B), exploiting the following gradient at a 0.4 mL min^−1^ flow rate: 30 min, 5 to 46% B; 2 min, 100% B, 10 min, 100% B (isocratic), 3 min, 5% B. The initial analysis employed ESI-MS detection in negative ion mode ([M-H]^−^) between 100 and 1000 mz and simultaneous detection at 280 nm using the PDA detector. Chromatograms were visualized, and peak areas were integrated with the LabSolutions 5.123 software. The Mass Profiler Professional 15.1 software (Agilent Technologies, Santa Clara, CA, USA) was used for the statistical analysis of identified compounds based on the obtained peak areas from UV chromatograms. ANOVA with a significance level of α = 0.05 was used to determine the compounds with statistically significant differences between the studied *Cuscuta* plants. Hierarchical clustering was performed to display the relationship between different samples based on compound relative abundance. Individual compounds were annotated based on comparisons of chromatograms, retention time, and the molecular ion mass, acquired in a preliminary study of *Cuscuta campestris* [27].

## 3. Results

### 3.1. Total Polyphenolics and Antioxidant Activity

**Figure 1:**
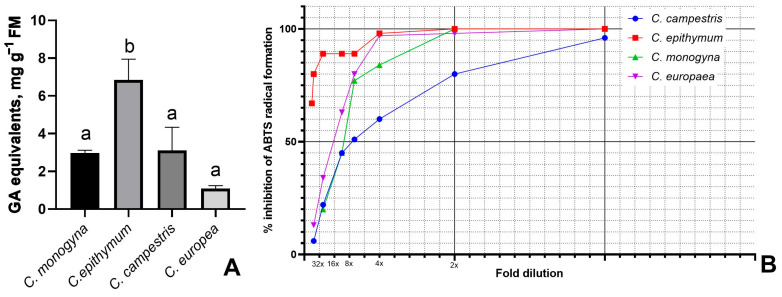
(**A**) Total polyphenolics in four *Cuscuta* species: mean values of different numbers of samples from wild populations ± SEM. Different letters indicate significant differences at *p* ≤ 0.05. (**B**) ABTS radical scavenging activity of 100% (*v*/*v*) methanolic extracts at different dilutions.

The number of phenolic compounds contained in the plant extracts is likely to be relevant to their antioxidant potential [28]. Therefore, the total concentration of polyphenolics was estimated, and mean values for each species are presented as gallic acid (GA) equivalents (Figure 1A). The highest concentration was found in *C. epithymum*, reaching up to 7.2 mg g^−1^ fresh weight, followed closely by *C. campestris* and *C. monogyna* with approximately three times lower concentrations. The lowest polyphenolic content was found in *C. europaea*. The samples with the highest polyphenolic concentration within each *Cuscuta* species were chosen for antioxidant activity measurements.

Antioxidant activity, expressed as the inhibition of ABTS radical formation, is presented in Figure 1B. The experimental data distinguished extracts of *C. epithymum* and *C. europaea*, which reached 98% inhibition at only 4-fold dilution. In comparison, the calculated values for *C. campestris* and *C. monogyna* at this concentration were 60% and 84%, respectively. However, all samples except *C. campestris* showed 100% inhibition of the initial, non-diluted extract, demonstrating their antioxidant potential in a concentration-dependent manner. The best antioxidant capacity was established for *C. epithymum*, as it retained high levels of inhibition (67%) even at a 32-fold dilution.

### 3.2. Flavonoid Content of Cuscuta Campestris in Relation to the Host Species

**Figure 2:**
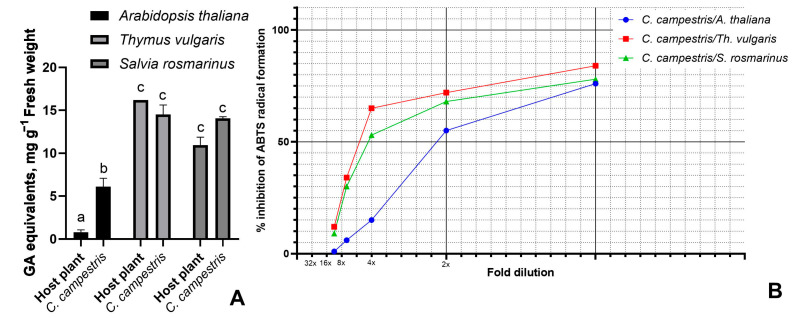
(**A**) Total polyphenolics of *Cuscuta campestris* and its host species, mean values ± SEM (n = 3). Different letters indicate significant differences at *p* ≤ 0.05. (**B**) ABTS radical scavenging activity of 100% (*v*/*v*) methanolic extracts at different dilutions.

*Cuscuta campestris* was chosen to determine the influence of the host species on the antioxidant activity despite the comparatively low polyphenolic content and antioxidant activity. This choice was governed by the wide distribution of the species, its broad host species range [29], the facility of growth, and the availability of an in-house sustained line. After the preliminary screening of different hosts and based on previous results [30], *A. thaliana* and two aromatic plants—rosemary and thyme—were selected for this study, ensuring the similar growth of the parasite. The phenolic concentrations were measured simultaneously in the host and the parasite (Figure 2A). The extracts of *Th. vulgaris* and *C. campestris*, grown on this host, showed the highest values of 16.2 and 14.5 mg g^−1^ GA equivalents. Comparable results were obtained for *S. rosmarinus*, where the polyphenolic concentration of the parasite was even higher than in the host. The extract of *A. thaliana* was distinguished by the lowest measured values, while the *C. campestris* grown on it had an approximately 8-fold higher concentration, highlighting the ability of the parasite to synthesize its own secondary polyphenolics. In conformity with the polyphenolic concentrations, the extracts of *C. campestris* differed in their ABTS scavenging activity (Figure 2B), with similar results on hosts *Th. vulgaris* and *S. rosmarinus* and much lower activity on host *A. thaliana*.

**Figure 3:**
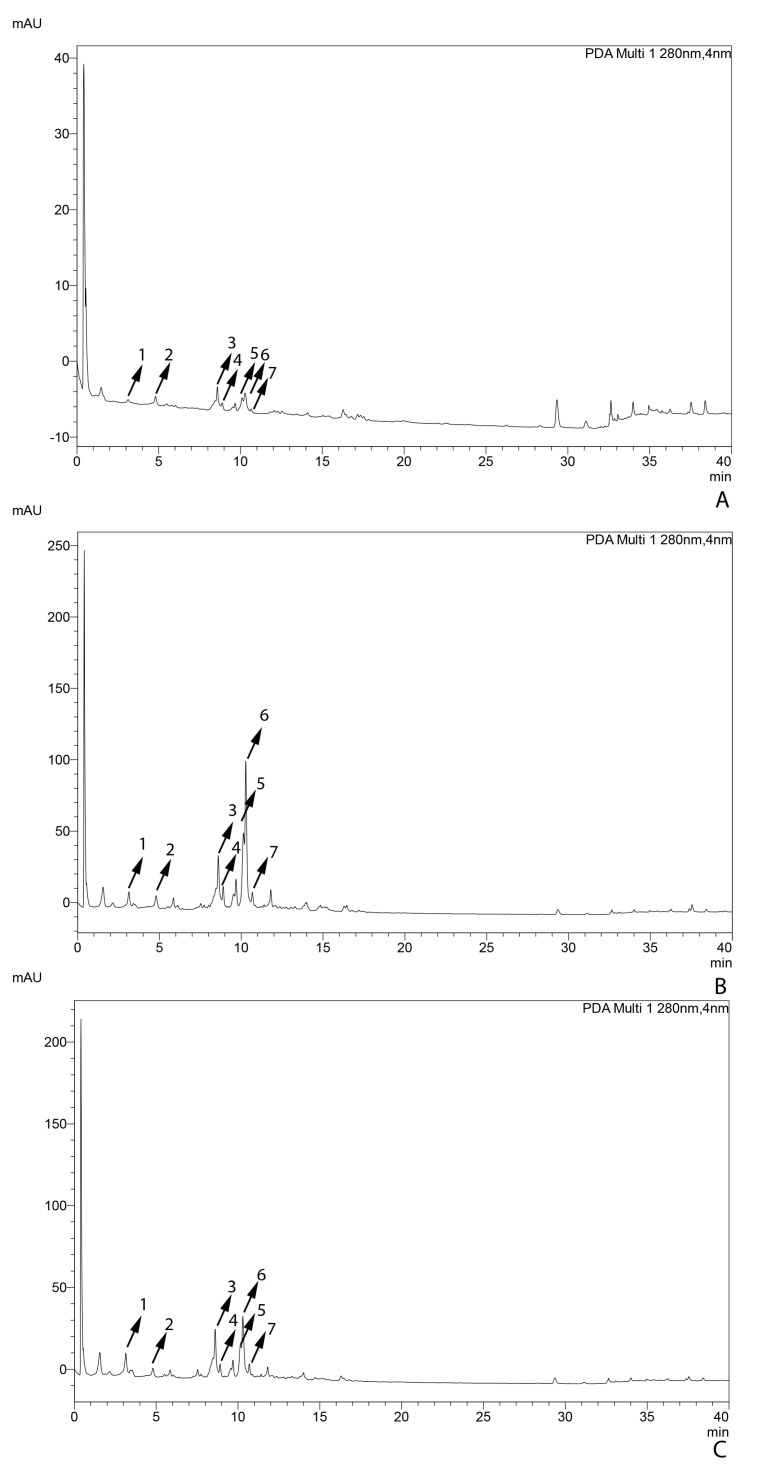
HPLC-PDA chromatograms of *C. campestris* grown on *Arabidopsis thaliana* (**A**), *Thymus vulgaris* (**B**), and *Salvia rosmarinus* (**C**). Numbers correspond to compounds in Table 1.

The flavonoid composition of *C. campestris*, grown on different hosts, was investigated through HPLC-MS. The resulting chromatograms from the PDA detector are shown in Figure 3 and Supplementary Figure S1. Arrows and the corresponding numbers indicate the compounds detected in these *Cuscuta* samples based on retention time and MS data (Table 1). A total of seven compounds were annotated. Analysis using ANOVA showed that six of the seven compounds varied statistically significantly (*p* < 0.05) among the three *Cuscuta* samples on different hosts, including chlorogenic acid, kaempferol-3,7-*O*-diglucoside, hyperoside, isoquercitrin, astragalin, and isorhamnetin-7-glucoside.

**Figure 4:**
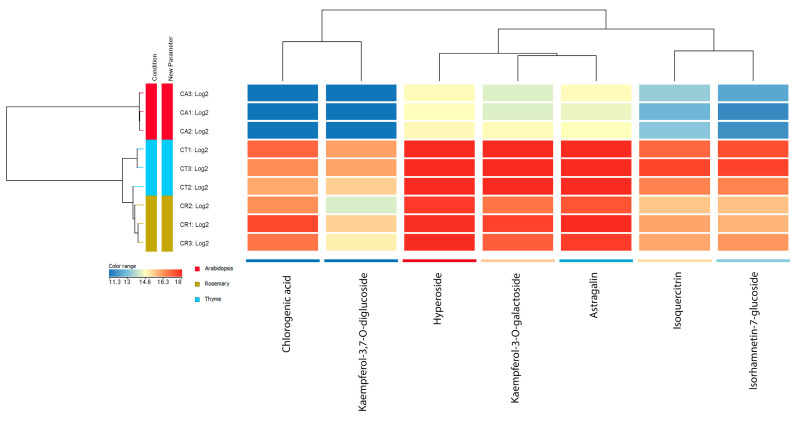
Hierarchical clustering of *C. campestris* samples grown on different hosts and the 7 flavonoid compounds. The dendrogram on the left shows the separation of samples into three main groups according to the host. The dendrogram at the top shows the relationship of the 7 compounds that varied significantly between samples based on their content in the samples.

The cluster analysis showed a separation based on the host species (Figure 4). According to clustering by samples, there was greater similarity between *Cuscuta* plants grown on *Th. vulgaris* and *S. rosmarinus*, while those on Arabidopsis were characterized by the greatest differences.

Higher flavonoid compound content was found in *Cuscuta* using thyme and rosemary as hosts. In addition, chlorogenic acid and kaempferol-3,7-*O*-diglucoside were absent from the composition of *Cuscuta* samples grown on Arabidopsis. A comparison between thyme and rosemary host samples showed that, on average, more flavonoid compounds (kaempferol-3,7-O-diglucoside, hyperoside, kaempferol-3-*O*-galactoside, astragalin, isoquercitrin, and isorhamnetin-7-glucoside) were present in the samples grown on thyme, whereas, in the presence of rosemary as host, higher average content for the hydroxycaffeic acid derivative chlorogenic acid was observed (Figure 4). These results indicate the differences in flavonoid composition even when comparing laboratory-grown *C. campestris* plants infecting hosts of the same family (Lamiaceae). This suggests the existence of much greater diversity and variation in the flavonoid profile of wild populations of *Cuscuta.* Host effects in wild populations are much more difficult to study because of the large number of hosts and the effects of multiple environmental factors that cannot be controlled.

## 4. Discussion

The relationship between antioxidant activity and polyphenolic concentration in plant extracts has been widely documented in the literature. Jafari et al. [31] specifically investigated this correlation in methanolic extracts of the aerial parts of three *Cuscuta* species. Two of these, *C. campestris* and *C. monogyna*, were included in the present study. Their analysis, using HPLC, revealed significant variations in phenolic retention, detecting approximately 16 metabolites, including gallic acid, catechin, caffeic acid, quercetin, coumarin, carvacrol, and vanillin. Among the species studied, *C. monogyna* exhibited the highest concentration of phenolic compounds (49.59 mg g^−1^ GA equivalents), followed by *C. campestris* (~40 mg g^−1^). This study also assessed the antioxidant activity of the extracts, with *C. monogyna* again demonstrating the highest antioxidant potential [31]. Our experimental results align with this, most notably in *C. epithymum*, which exhibited both the highest total polyphenolic content and the greatest antioxidant potential. A notable discrepancy was observed in *C. europaea*, which demonstrated strong antioxidant potential despite its low polyphenolic content. Although most studies relate the antioxidant potential in *Cuscuta* extracts to polyphenols, and flavonoids in particular [14,31], other phytochemicals, like carotenoids [32], may also contribute to this [33].

Given the parasitic nature of *Cuscuta* species and their nonspecific relationships with host plants, researchers have increasingly focused on characterizing plant–plant metabolic exchanges. Kumar et al. conducted a metabolic profiling study on *C. campestris* grown on three different host plants, revealing significant variations in both primary and secondary metabolite levels. The highest polyphenolic concentrations were detected in the haustoria—the specialized structures facilitating host contact—originating from the parasite’s stem [34]. Similarly, analysis of *Cuscuta*–host interactions through metabolomic and transcriptomic analyses indicated that 72 h after infection, phenolic acids were among the most abundant metabolite groups exchanged between the host and parasite [35].

Thyme (*Thymus vulgaris*) and rosemary (*Salvia rosmarinus*) are well-known medicinal plants with strong antioxidant, antibacterial, and anti-inflammatory properties, widely used in traditional medicine. Their health benefits are primarily attributed to their high polyphenolic content. Thyme contains flavonoids such as apigenin and luteolin in various glycoside forms, while rosemary is rich in flavonoids, including flavones (e.g., diosmin), flavonols (e.g., kaempferol, quercetin, and their glycosides), and rosemary-specific compounds like rosmarinic acid [36,37]. In contrast to the diverse polyphenolic and volatile compound profiles of thyme and rosemary, the model plant *Arabidopsis thaliana* has a simpler phenolic composition, primarily consisting of quercetin, kaempferol, and their glycosides [38]. In alignment with this, the extracts of *C. campestris*, parasitizing the two aromatic plants, showed much higher polyphenolic content and respective antioxidant activity. These results indicate that the parasitic plant not only has its own polyphenolic synthesis, as shown in the *Arabidopsis* host, but also obtains polyphenolics from aromatic plants as hosts. So far, it is hard to say how efficient the secondary metabolism in these parasites is. Further, flavonoids obtained from the host plant may undergo additional modifications. For example, glycosylation and oxidation have been observed in *C. campestris* and *C. kotchiana*, leading to the formation of metabolites distinct from those in the host plant [15].

Further studies have explored how host plant species influence the bioactive compound profile of *C. campestris*. *When grown on* herbaceous hosts, it exhibited higher concentrations of bioactive compounds such as flavonoids, phytosterols, and polyphenols. Among the tested hosts, thyme supported the highest levels of bergenin and total phenols, and its associated *Cuscuta* extract displayed some of the strongest antioxidant activities [39]. A similar study on *C. reflexa* showed host-dependent variations in rutin, quercetin, catechin, gallic acid, and vanillic acid concentrations [40]. Apparently, in laboratory conditions, as in our results, the diversity and content of flavonoids are much lower than in naturally grown populations. However, it was still demonstrated that the flavonoid profile of these parasitic plants was strongly affected by the host species, and even within the same family (Lamiaceae in this case), different hosts led to differences in the parasite.

Overall, members of the genus *Cuscuta* are generalists, meaning they have a broad host range from a taxonomical perspective [29], but they also infect multiple hosts simultaneously [41]. This not only ensures adequate nutrient flow but also the transfer of molecules with diverse functions. As previously established [42], the haustoria enable intense macromolecular exchange. By forming an extensive network on infected plants, a single dodder individual may benefit from receiving key signals of potential threats like herbivores [43] and may acquire defensive molecules. This is clearly a strong adaptive advantage. Considering flavonoids, our previous results showed a species-specific profile, somehow affected by the hosts [27]. In the present study, it was found that in *C. campestris*, the role of the host mainly involves the relative abundance of individual compounds and the overall quantity of polyphenolics, rather than dramatic differences in the profile. However, when grown on a single host as in this case, the diversity of flavonoids seems to be much lower than in samples collected in the wild from multiple hosts [27]. Especially for dodders, the accumulation of these compounds may be of crucial importance due to their antioxidant and photoprotective role in the plant kingdom [44]. Along with the unique carotenoid cycle [32], they would be crucial because of the direct exposure to light of this parasite.

## 5. Conclusions

The present study demonstrated that parasitic plants of the genus *Cuscuta* possess high polyphenolic content and antioxidant potential. These features are species-dependent but also strongly depend on the host species, as some host plant species further enhance the polyphenolic content of the parasite. While growing equally well on the flavonoid-poor model host *Arabidopsis thaliana* and the aromatic hosts—thyme and rosemary—quantitatively, the number of polyphenols was much higher in the parasite when parasitizing the later hosts. This is also evidence that these parasites do not rely on their hosts only for water and nutrients but also on protective molecules, such as flavonoids, which are important in antioxidant defense and photoprotection. Their ability to simultaneously parasitize individuals from different taxonomic groups may be an important feature for acquiring compounds with diverse structures and functions, enabling metabolic plasticity and better adaptability to environmental factors. However, a more thorough study, encompassing additional secondary metabolites, along with more accurate quantitative analyses, would further help us understand the chemical basis of the antioxidative potential of *Cuscuta* extracts. Another important question not addressed here is the effect of *Cuscuta* parasitism on the flavonoid (and, overall, secondary metabolite) content in the host plants, which would further contribute to understanding the phenomenon of plant-to-plant parasitism.

## Figures and Tables

**Table 1:** Compounds, annotated in *Cuscuta campestris* by HPLC-MS. t_R_—retention time. [M-H]^−^ m/z—mass-to-charge ratio of the molecular ion in negative ion mode.

Peak	t_R_ (min)	Identity	[M-H]^−^ m/z
1	3.15	Chlorogenic acid	353.0858
2	4.85	Kaempferol-3,7-O-diglucoside	609.1439
3	8.59	Quercetin-3-O-galactoside (Hyperoside)	463.0853
4	8.94	Kaempferol-3-O-galactoside	447.0902
5	10.17	Quercetin-3-O-glucoside (Isoquercitrin)	463.0852
6	10.36	Astragalin	447.0902
7	10.66	Isorhamnetin-7-glucoside	477.1005

## Data Availability

The data are contained within the article or Supplementary Materials.

## Supplementary Materials

The following supporting information can be downloaded at https://www.mdpi.com/article/10.3390/antiox14070761/s1.

## Abbreviations

The following abbreviations are used in this manuscript: ABTS—2,2′-azino-bis(3-ethylbenzothiazoline-6-sulfonic acid; ANOVA—analysis of variance; dH_2_O—distilled water; ESI—electrospray ionization; GA—gallic acid; HPLC—high-performance liquid chromatography; MS—mass spectrometry; PDA—photodiode array; SEM—standard error of the mean.
