# Supplementary material for: The Antioxidant Properties of Extracts of Cuscuta spp. Depend on the Parasite and the Host Species
Source: Antioxidants (Basel). 2025 Jun 20;14(7):761. doi: 10.3390/antiox14070761 (PMC12291675; doi:10.3390/antiox14070761)
Supplement: Supplementary file 1 [file antioxidants-14-00761-s001.zip › antioxidants-3642341-supplementary.pdf]

==== Shimadzu LabSolutions Multi-Chromatogram ====

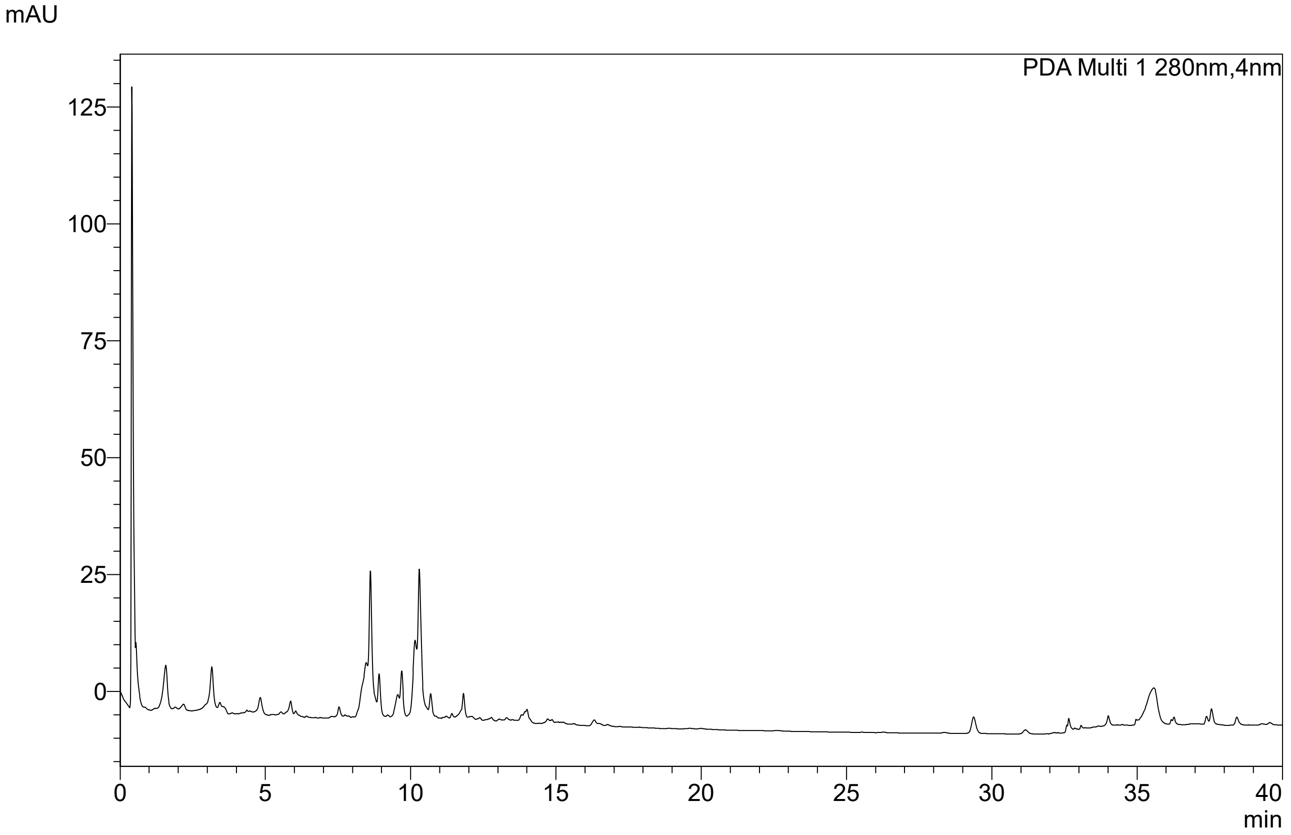

Cuscuta campestris, rosemary sample 1

==== Shimadzu LabSolutions Data Report ====

<Spectrum>

Line#:1 R.Time:0.000(Scan#:1)
MassPeaks:1360
RawMode:Single 0.000(1) BasePeak:191(5770)
BG Mode:None Segment 1 - Event 1

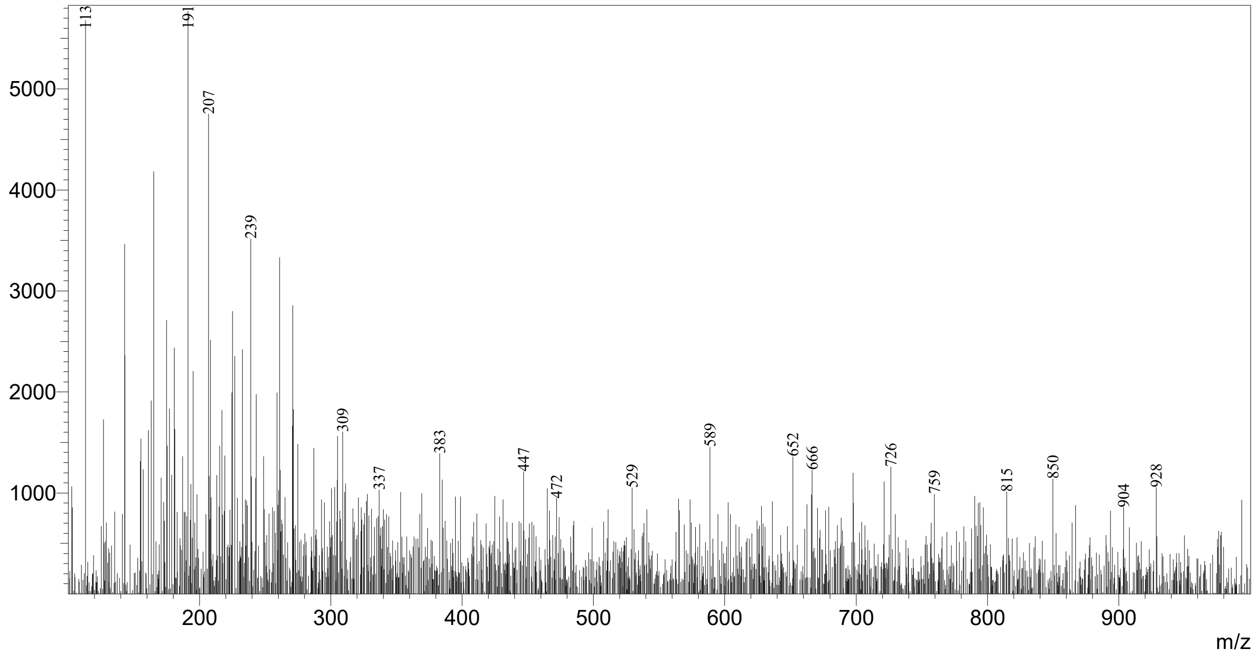

Cuscuta campestris, rosemary sample 1

==== Shimadzu LabSolutions Multi-Chromatogram ====

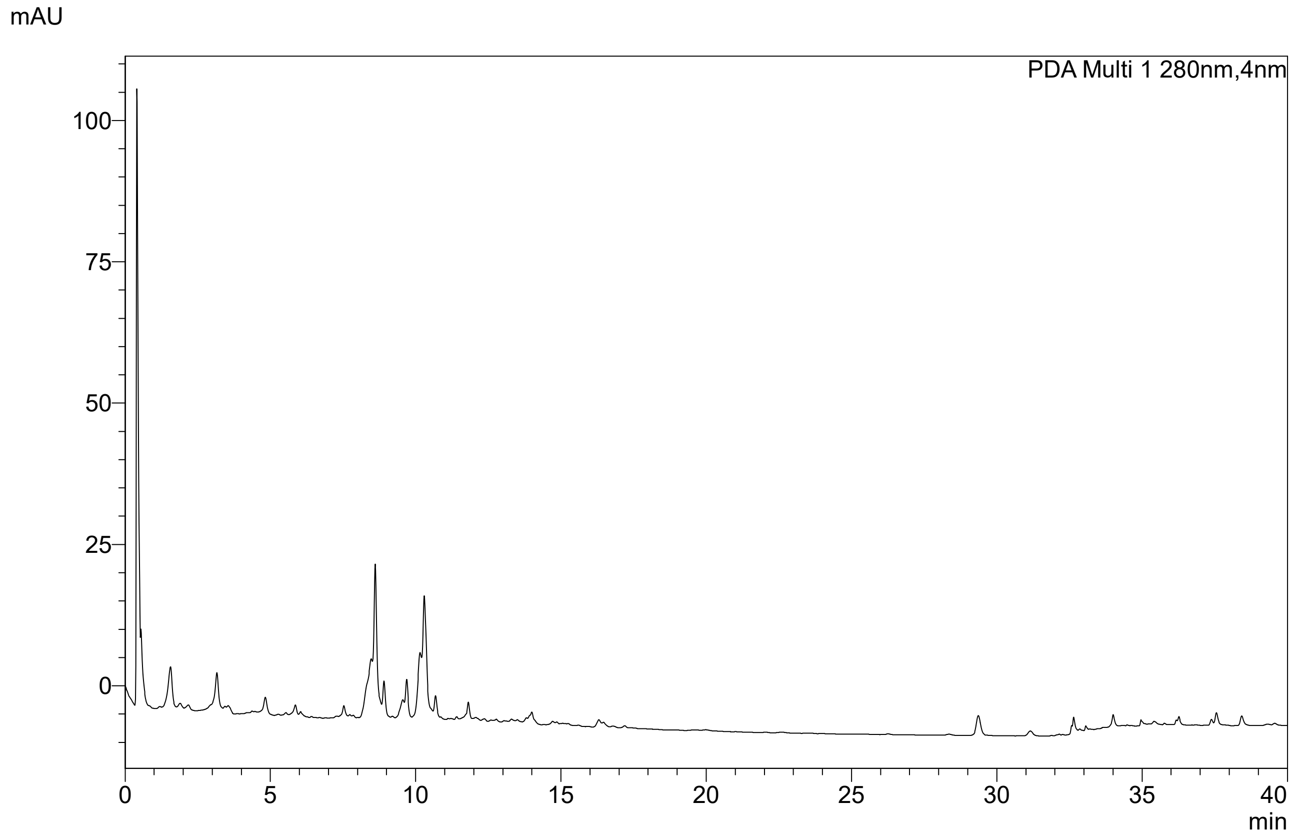

Cuscuta campestris, rosemary sample 2

==== Shimadzu LabSolutions Data Report ====

<Spectrum>

Line#:1 R.Time:0.000(Scan#:1)
MassPeaks:1347
RawMode:Single 0.000(1) BasePeak:271(7213)
BG Mode:None Segment 1 - Event 1

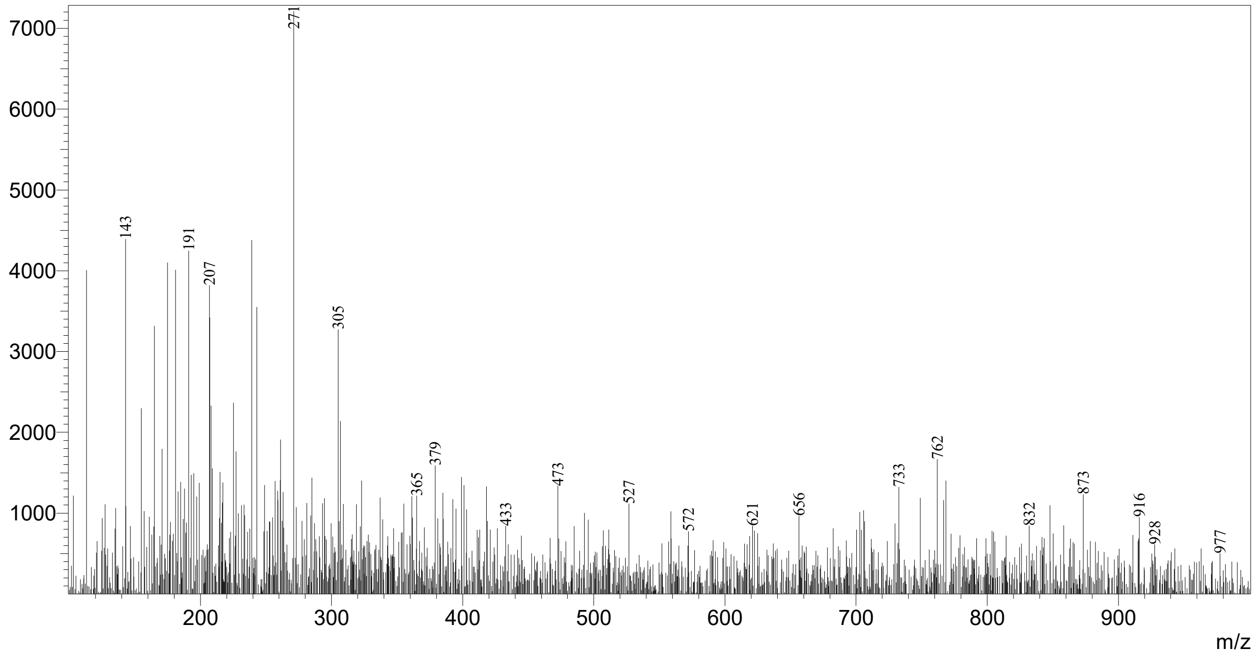

Cuscuta campestris, rosemary sample 2

==== Shimadzu LabSolutions Multi-Chromatogram ====

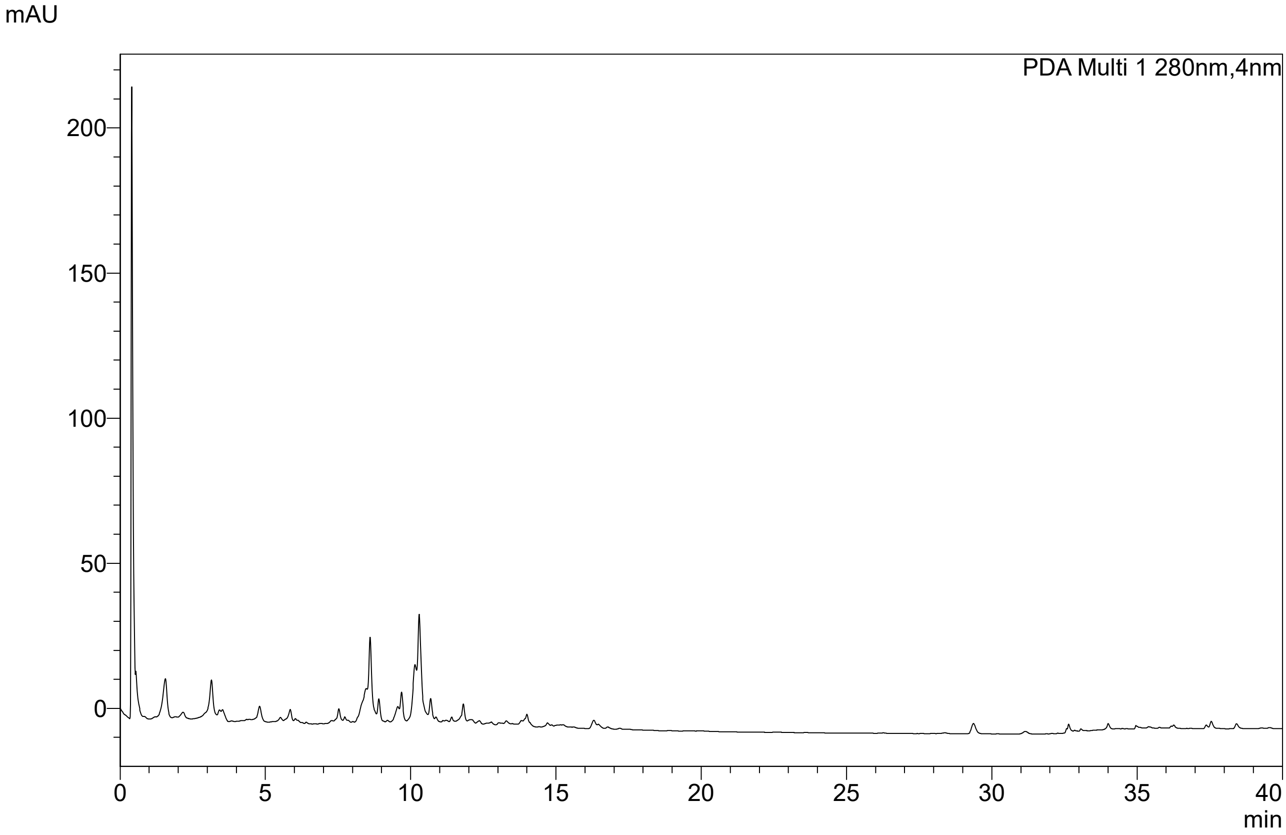

Cuscuta campestris, rosemary sample 3

==== Shimadzu LabSolutions Data Report ====

<Spectrum>

Line#:1 R.Time:0.000(Scan#:1)
MassPeaks:1354
RawMode:Single 0.000(1) BasePeak:207(4991)
BG Mode:None Segment 1 - Event 1

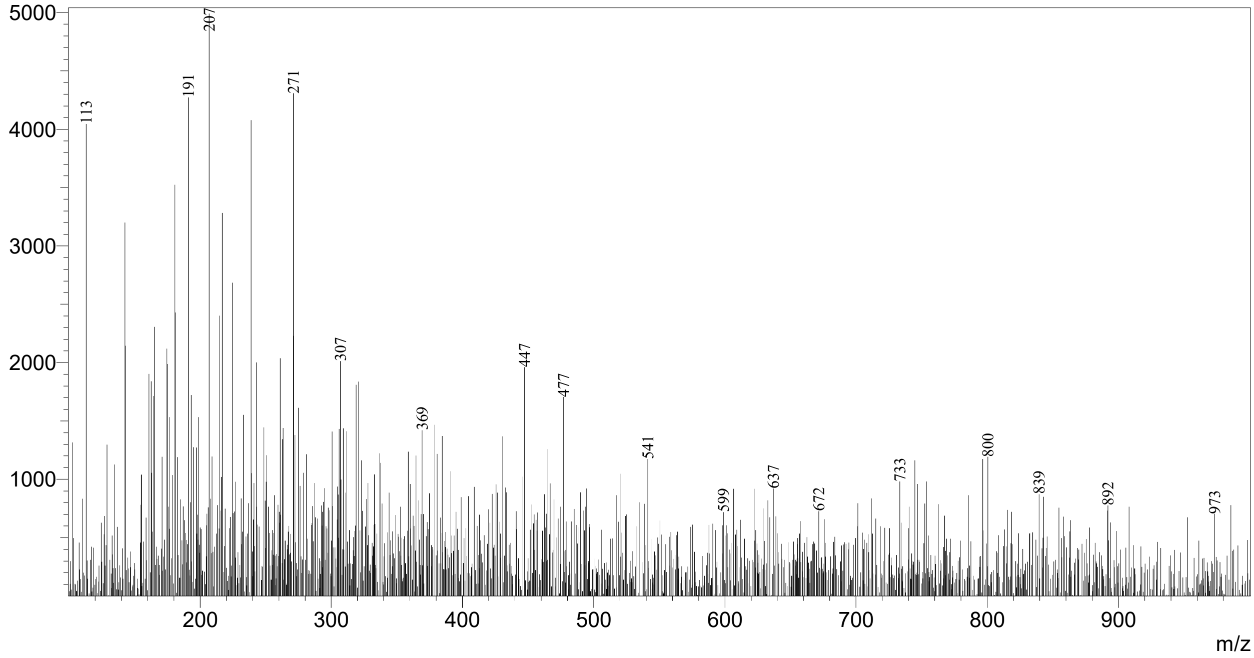

Cuscuta campestris, rosemary sample 3

==== Shimadzu LabSolutions Multi-Chromatogram ====

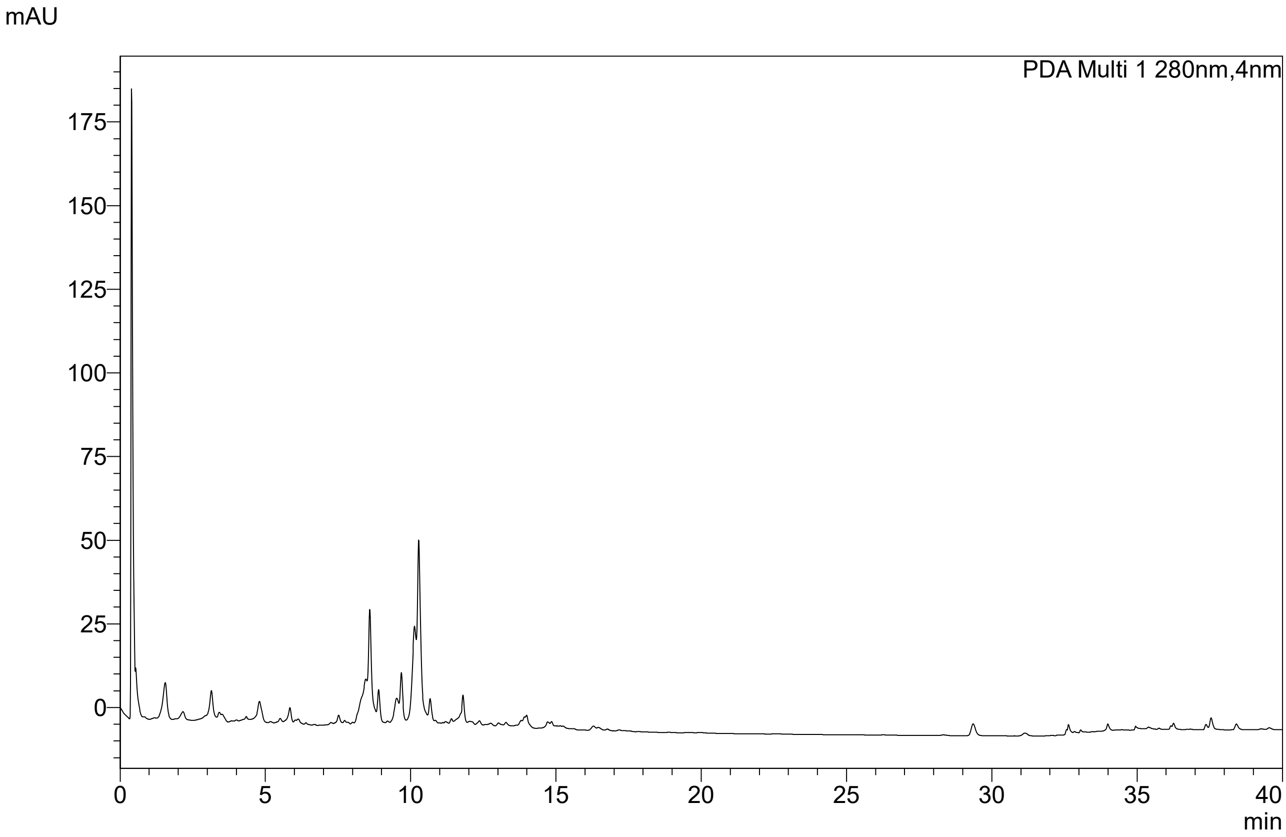

Cuscuta campestris, thyme sample 1

==== Shimadzu LabSolutions Data Report ====

<Spectrum>

Line#:1 R.Time:0.000(Scan#:1)
MassPeaks:1356
RawMode:Single 0.000(1) BasePeak:271(6092)
BG Mode:None Segment 1 - Event 1

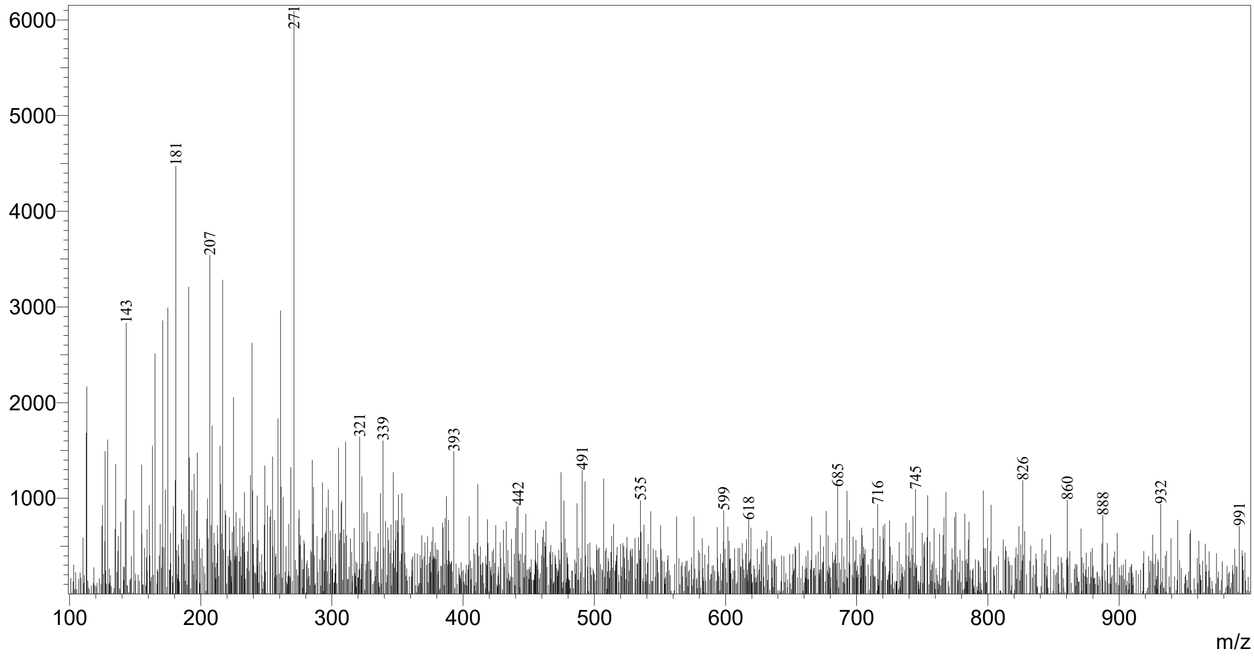

Cuscuta campestris, thyme sample 1

==== Shimadzu LabSolutions Multi-Chromatogram ====

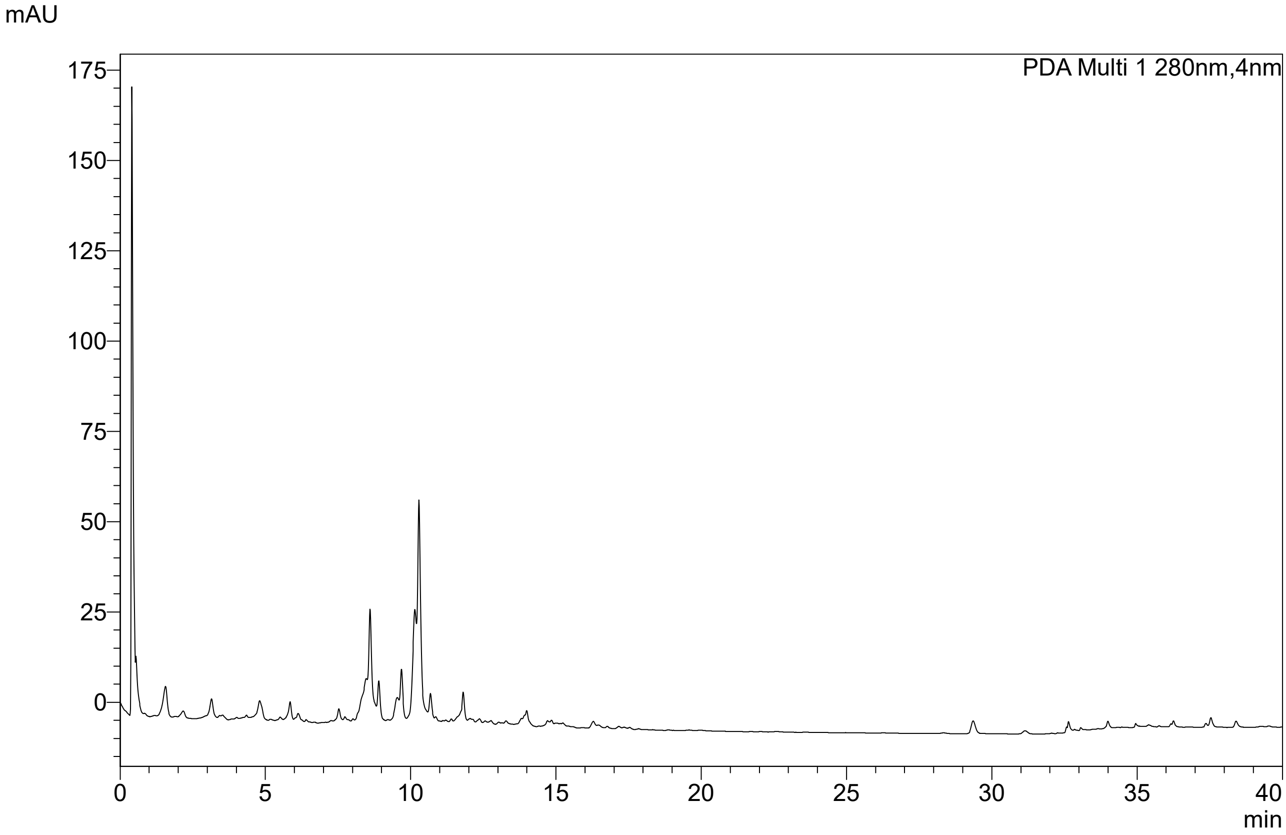

Cuscuta campestris, thyme sample 2

==== Shimadzu LabSolutions Data Report ====

<Spectrum>

Line#:1 R.Time:0.000(Scan#:1)
MassPeaks:1376
RawMode:Single 0.000(1) BasePeak:113(6580)
BG Mode:None Segment 1 - Event 1

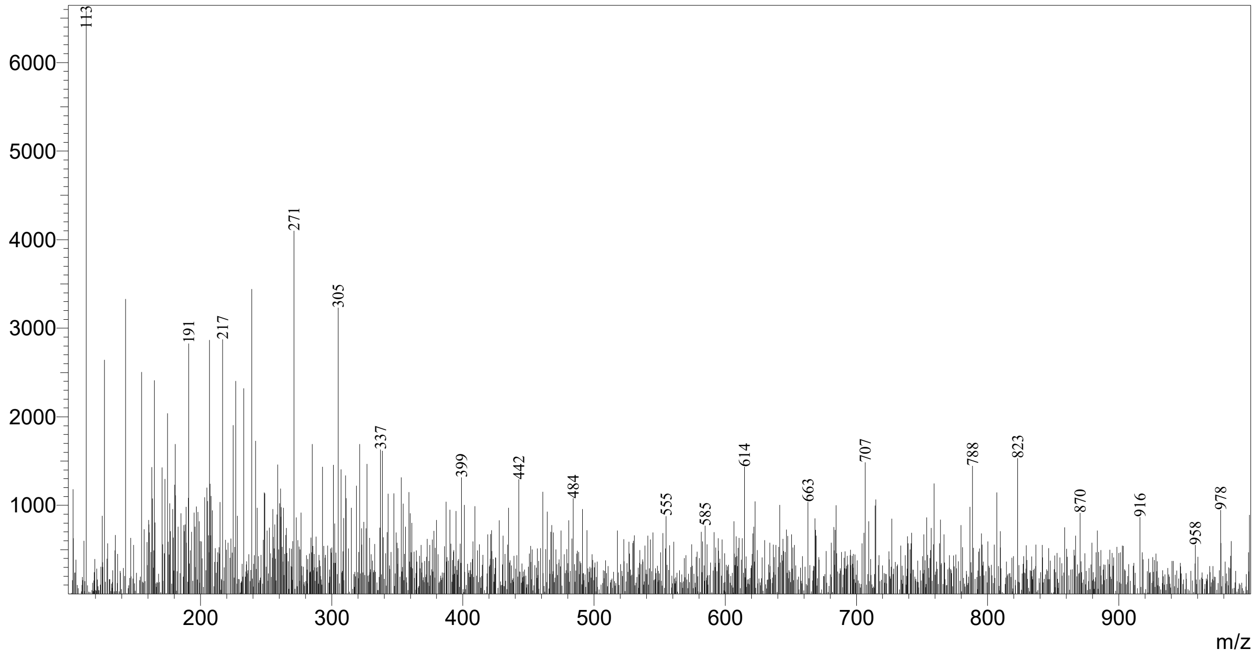

Cuscuta campestris, thyme sample 2

==== Shimadzu LabSolutions Multi-Chromatogram ====

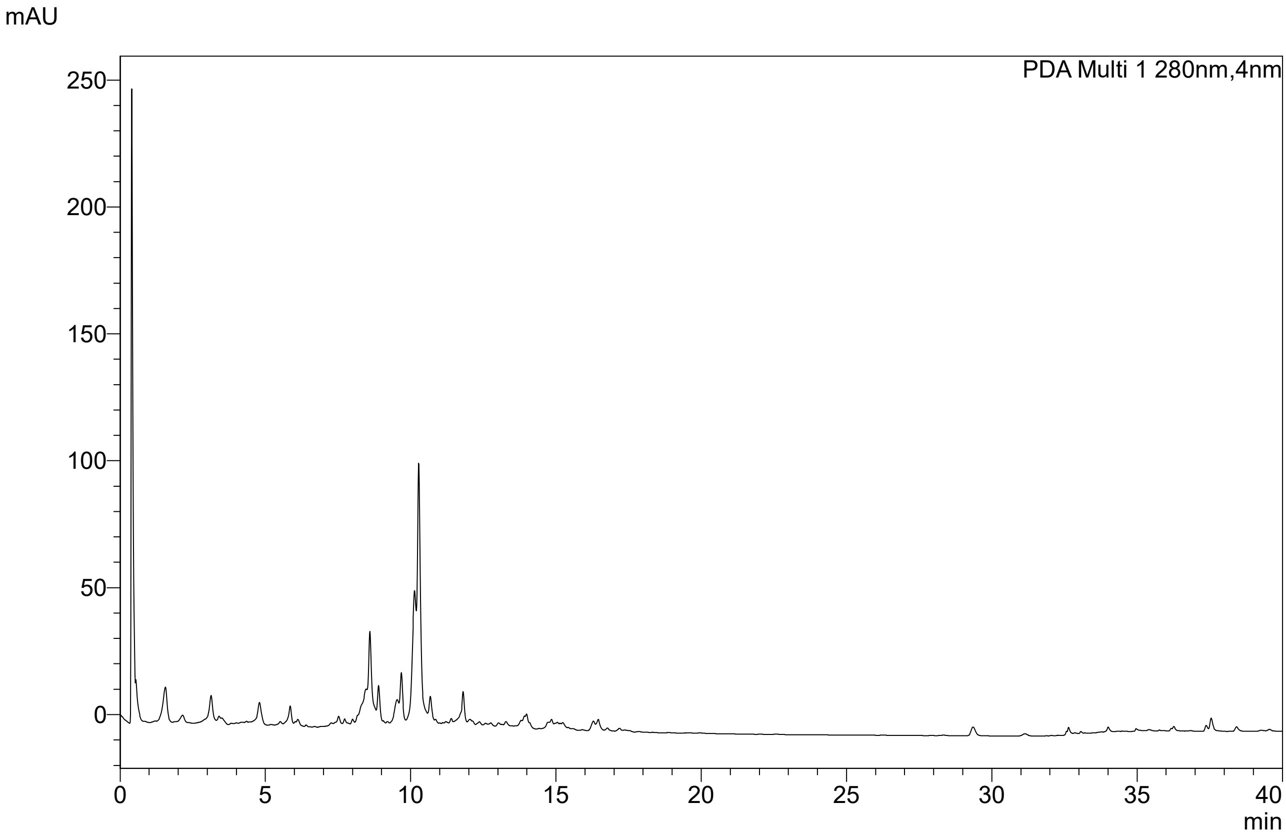

Cuscuta campestris, thyme sample 3

==== Shimadzu LabSolutions Data Report ====

<Spectrum>

Line#:1 R.Time:0.000(Scan#:1)
MassPeaks:1370
RawMode:Single 0.000(1) BasePeak:271(5144)
BG Mode:None Segment 1 - Event 1

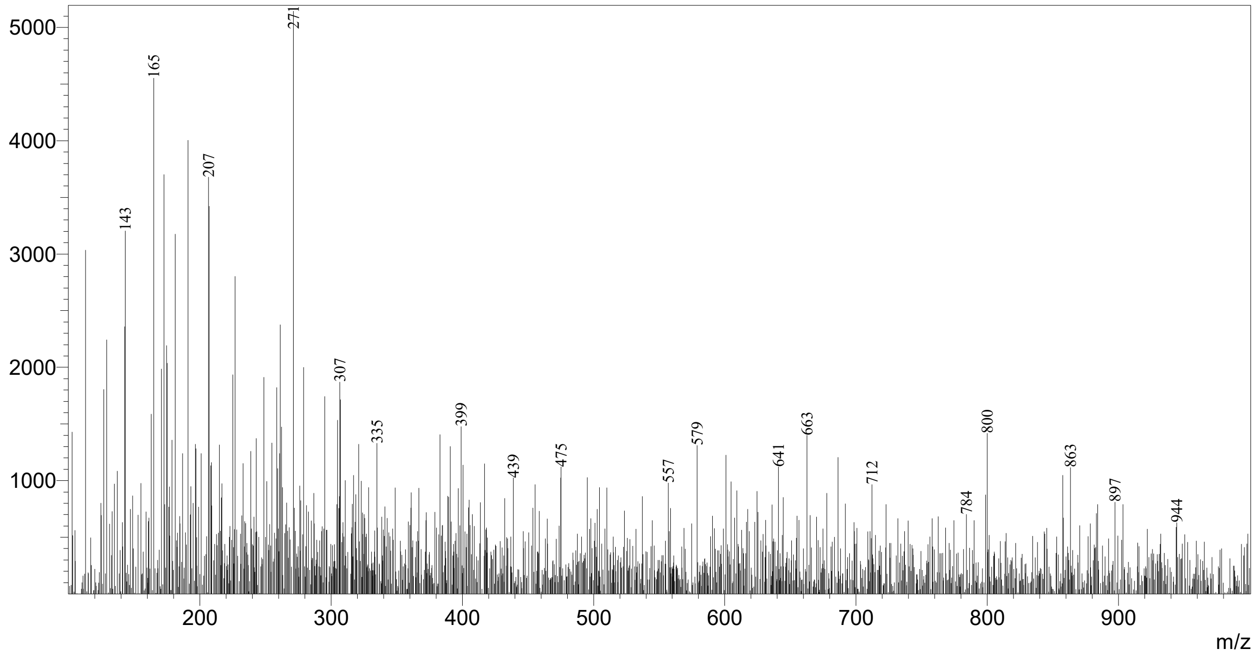

Cuscuta campestris, thyme sample 3

==== Shimadzu LabSolutions Multi-Chromatogram ====

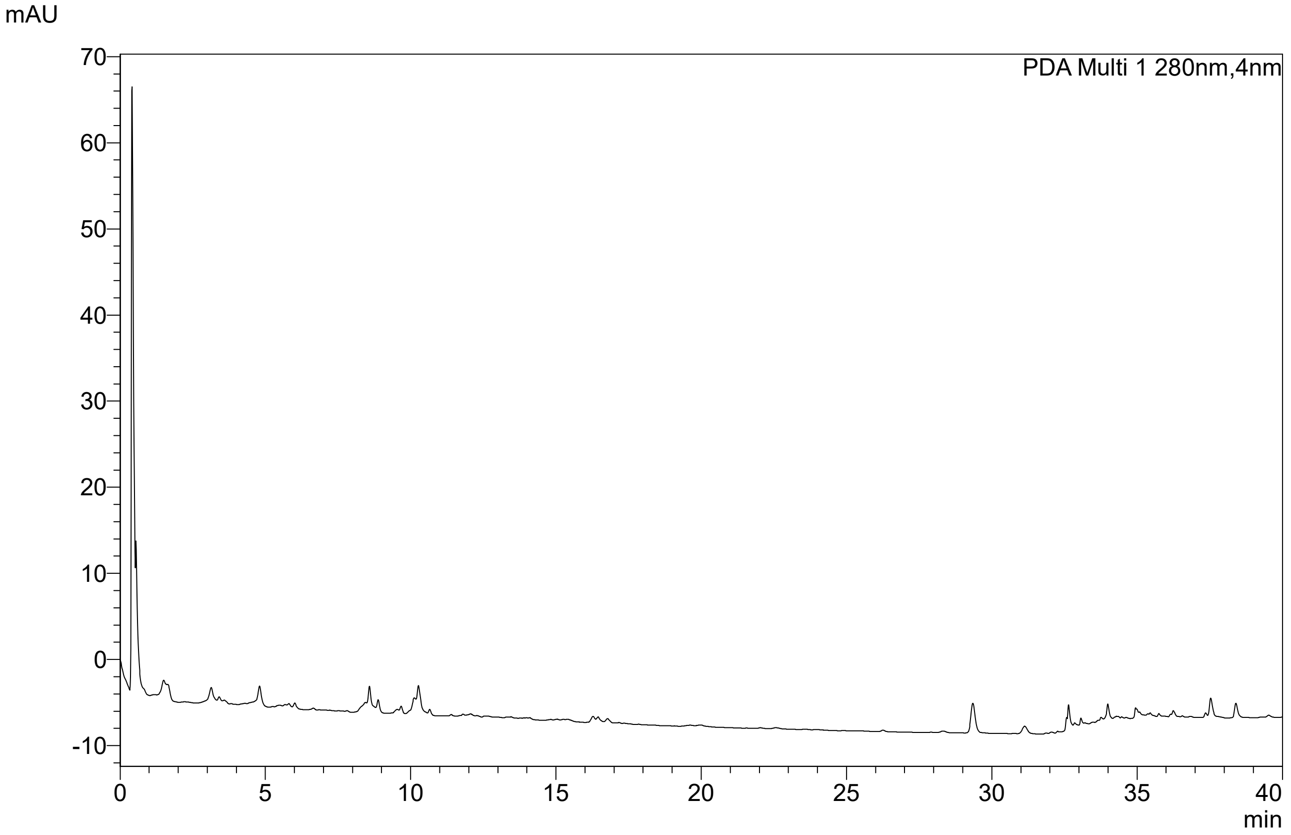

Cuscuta campestris, Arabidopsis sample 1

==== Shimadzu LabSolutions Data Report ====

<Spectrum>

Line#:1 R.Time:0.000(Scan#:1)
MassPeaks:1322
RawMode:Single 0.000(1) BasePeak:207(8433)
BG Mode:None Segment 1 - Event 1

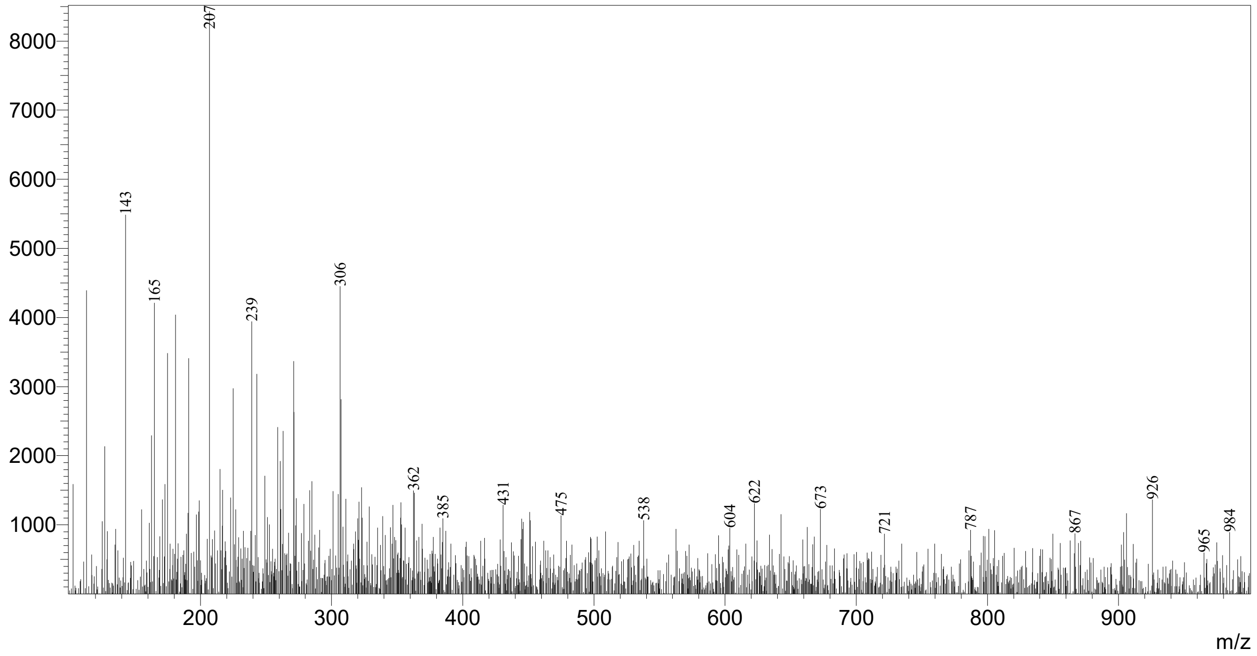

Cuscuta campestris, Arabidopsis sample 1

==== Shimadzu LabSolutions Multi-Chromatogram ====

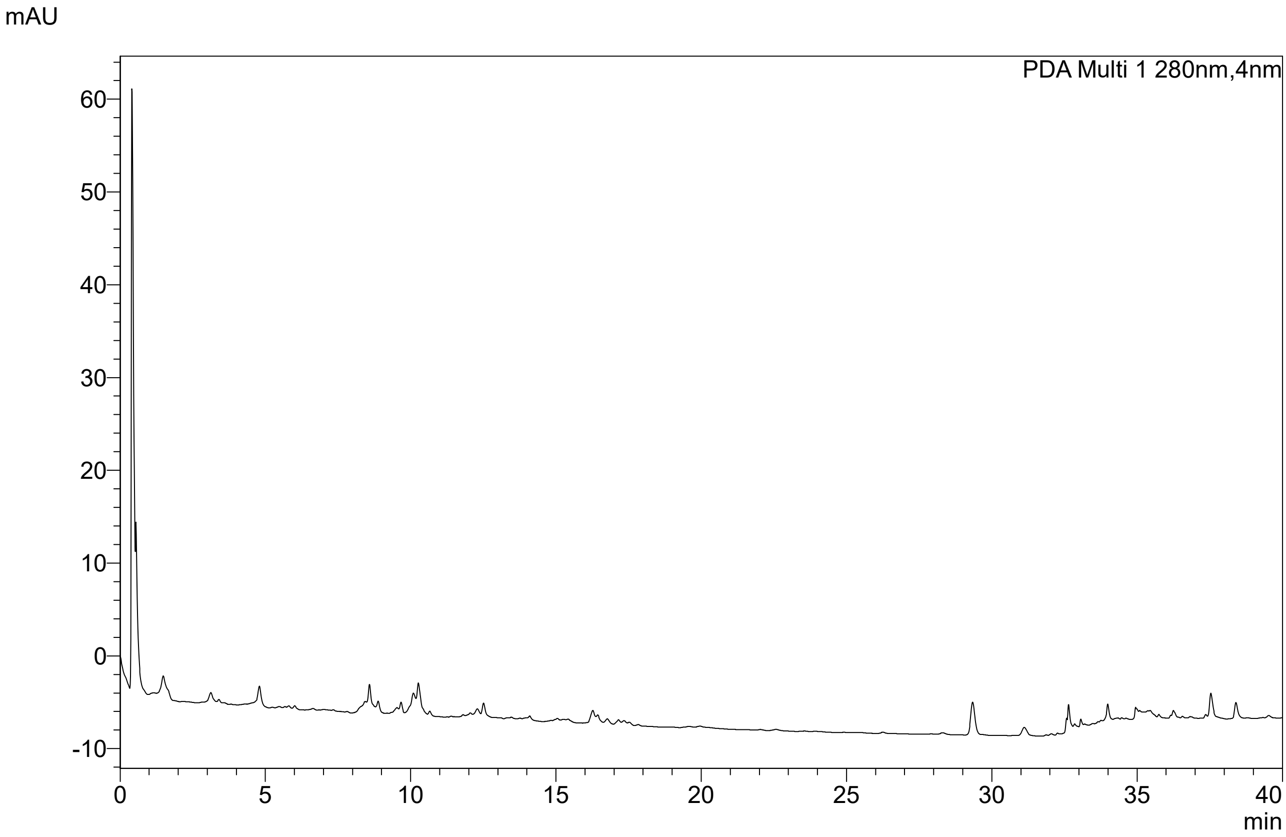

Cuscuta campestris, Arabidopsis sample 2

==== Shimadzu LabSolutions Data Report ====

<Spectrum>

Line#:1 R.Time:0.000(Scan#:1)
MassPeaks:1309
RawMode:Single 0.000(1) BasePeak:271(6268)
BG Mode:None Segment 1 - Event 1

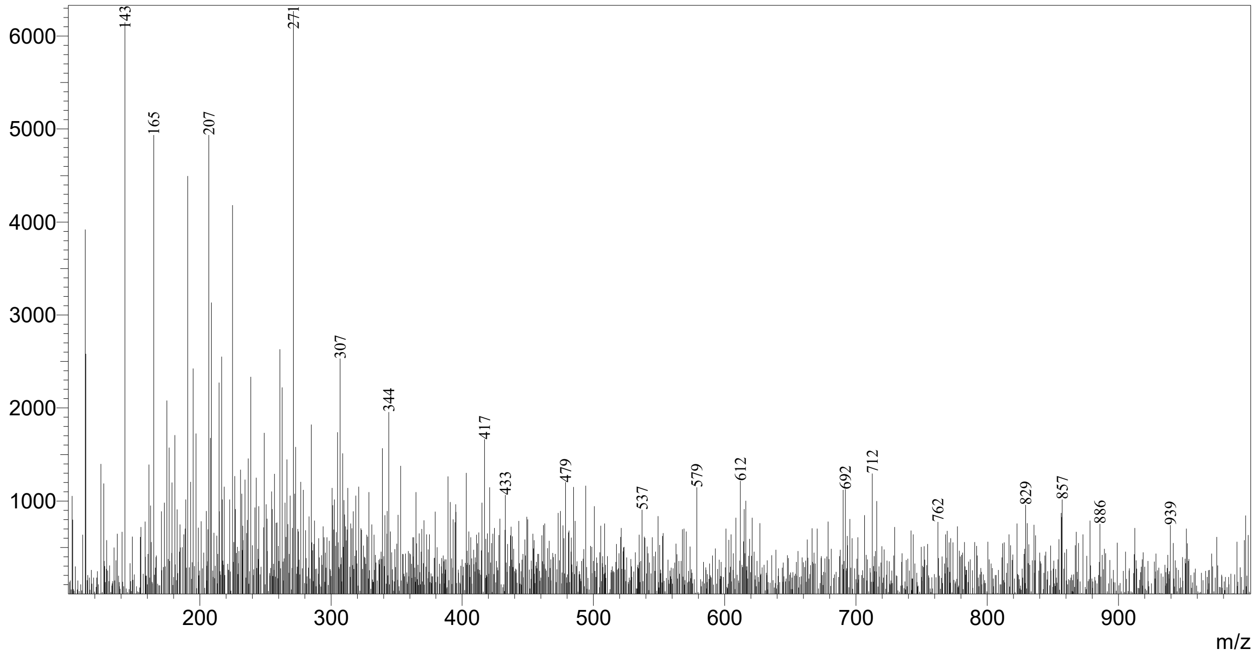

Cuscuta campestris, Arabidopsis sample 2

==== Shimadzu LabSolutions Multi-Chromatogram ====

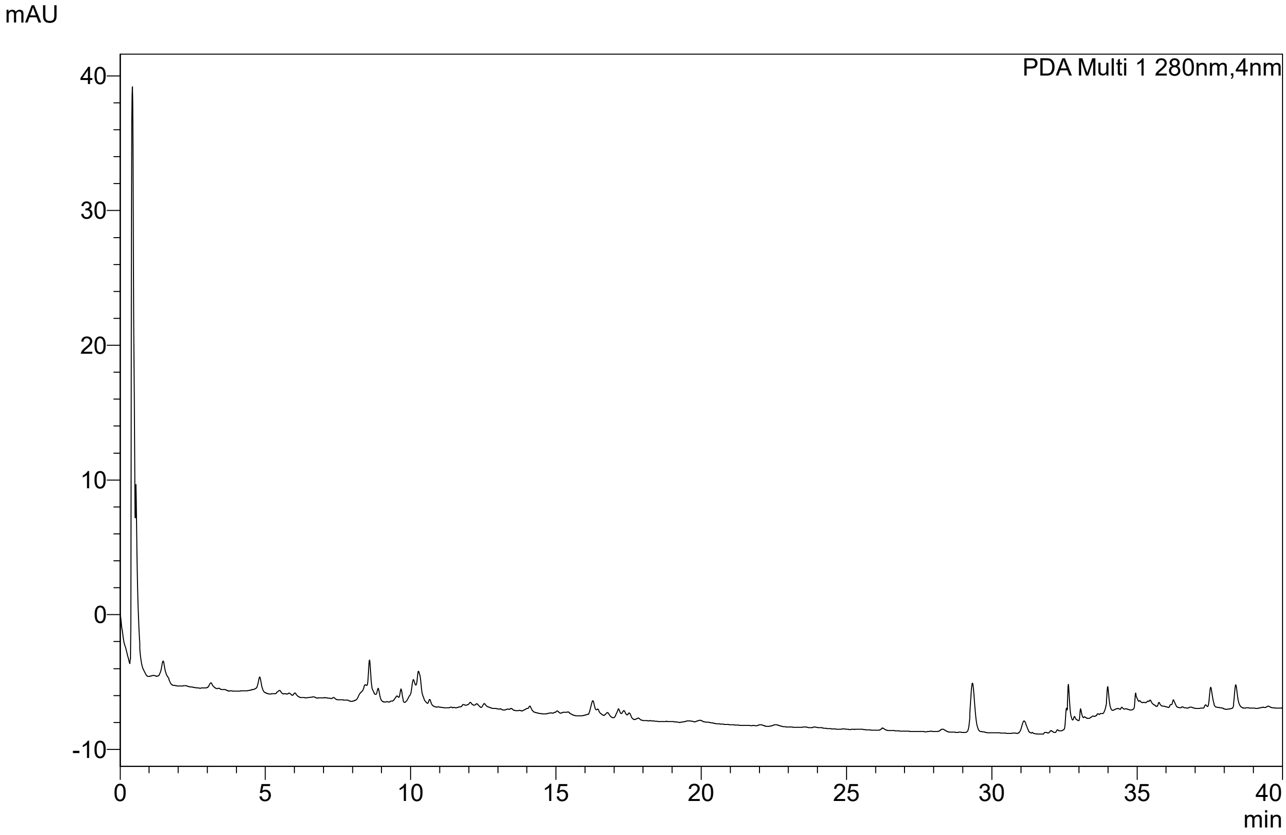

Cuscuta campestris, Arabidopsis sample 3

==== Shimadzu LabSolutions Data Report ====

<Spectrum>

Line#:1 R.Time:0.000(Scan#:1)
MassPeaks:1325
RawMode:Single 0.000(1) BasePeak:113(4496)
BG Mode:None Segment 1 - Event 1

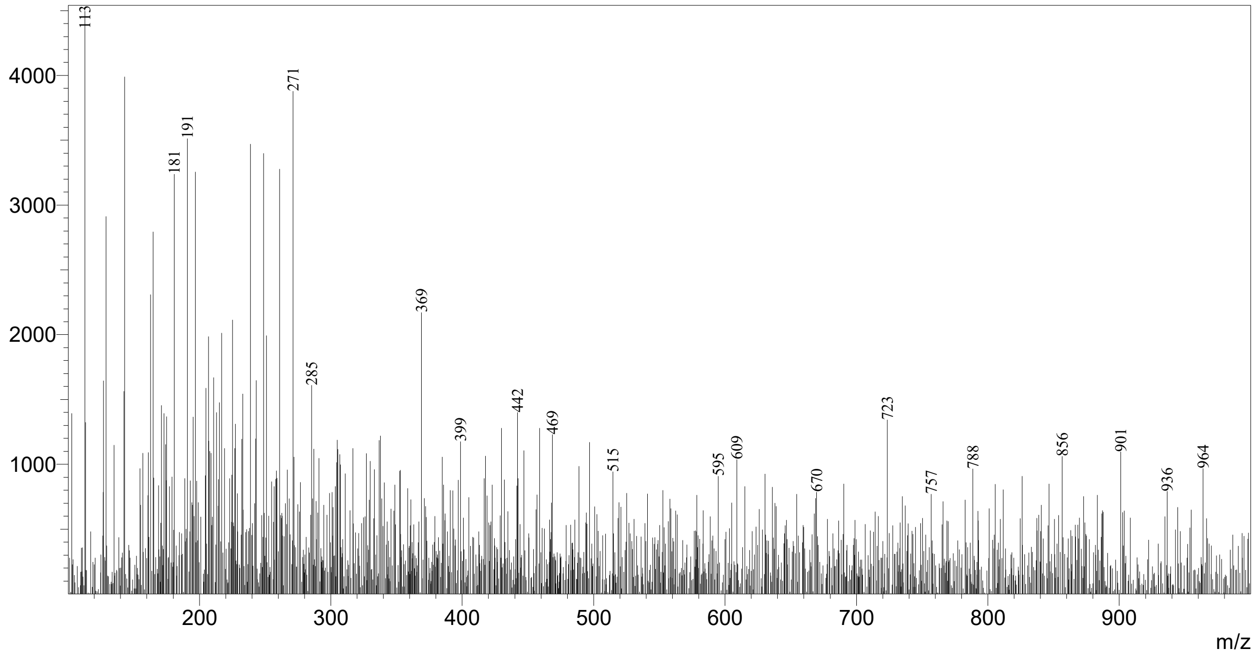

Cuscuta campestris, Arabidopsis sample 3
